# Single atom catalysis: a decade of stunning progress and the promise for a bright future

**DOI:** 10.1038/s41467-020-18182-5

**Published:** 2020-08-27

**Authors:** Sharon Mitchell, Javier Pérez-Ramírez

**Affiliations:** Department of Chemistry and Applied Biosciences, Institute for Chemical and Bioengineering, ETH Zurich, Vladimir-Prelog-Weg 1, 8093 Zurich, Switzerland

**Keywords:** Heterogeneous catalysis, Chemical engineering, Synthesis and processing

## Abstract

Controlling the hybridization of single atoms in suitable host materials opens unique opportunities for catalyst design, but equally faces many challenges. Here, we highlight emerging directions from the last, highly productive, decade in single-atom catalysis and identify frontiers for future research.

## Origins and evolution of single-atom catalysis

**Fig. 1:**
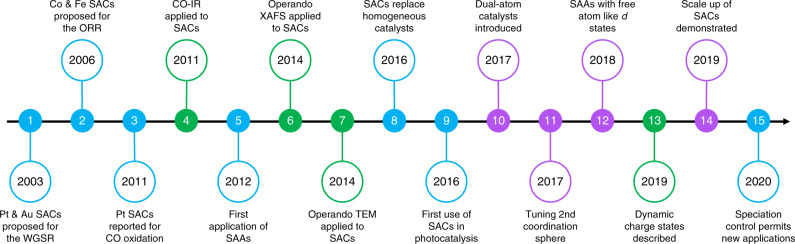
Progress in single-atom catalysis. Timeline showing landmarks (L = 1–15) leading up to and during the last decade of research on the synthesis (purple), characterization (green), and application (blue) of single-atom metal catalysts. ORR oxygen reduction reaction, WGSR water–gas shift reaction, SAA single-atom alloy, TEM transmission electron microscopy, XAFS X-ray absorption fine structure, CO-IR infrared spectroscopy of adsorbed CO.

The isolation of elements as atoms in chemically-distinct substances played fundamental catalytic roles, for example, in metalloenzymes, organometallic complexes, and open framework structures, long before this concept was extended to widely applied heterogeneous catalysts based on supported metals. In pioneering early work, Flytzani-Stephanopoulos et al. provided convincing evidence that ionic gold or platinum species strongly associated with the surface of ceria, and not the metal nanoparticles, were responsible for the activity observed in the water–gas shift reaction (Fig. 1, L1)^1^. Subsequently, Bashyam and Zelenay proposed that oxidized cobalt and iron species coordinated to nitrogen and oxygen in functionalized carbons deliver high performance in the electrochemical oxygen reduction reaction (Fig. 1, L2)^2^. After years of speculation about the catalytic role of single atoms of this group of elements, advances in experimental techniques made it possible to confirm the exclusive presence of isolated centers. In their landmark paper, Zhang et al. confirmed the high efficiency of platinum atoms supported on iron oxide for CO oxidation, introducing a new paradigm in heterogeneous catalysis (Fig. 1, L3)^3^. The first catalytic applications of single-atom alloys based on metal hosts followed shortly after (Fig. 1, L5)^4^. In just a decade, the topic of single-atom catalysis has become a highly transversal field of contemporary chemical research.

Early researchers quickly recognized the potential cost benefits of using atomically-dispersed species for precious metals, and improving the utilization became a central driver in the topic. As the area developed, the motivation also evolved. Comparative studies revealed that the strategy of using SACs does not apply to all applications and depends on the reaction requirements^5^. Another strength is the higher uniformity of potential active sites in heterogeneous catalysts based on single atoms compared to nanoparticles. The well-defined nature is attractive for both defining structure–function relationships and computational modeling. Furthermore, the close structural resemblance to molecular complexes provides a bridge between homogeneous and heterogeneous catalysis.

On the 10-year journey since the consolidation of the topic, single-atom catalysis has crossed the periodic table. The diversity of host materials has also expanded. While metal oxides were initially most widely studied, tailored carbons have superseded all other types in the last years. The scope of applications has diversified, with electrochemical conversions growing in emphasis and promising findings emerging in photocatalysis. Recognition of the limitations of existing techniques has also prompted increased precision in the characterization, demanding the use of multiple complementary methods as well as investigation under operando conditions (Fig. 1, L4, L6, and L7)^6^. Here, we highlight some of the main directions developing from the intense efforts of the scientific community as well as frontiers in the design of SACs.

## Tailored local environments

During the discovery of the first SACs based on late-transition metals, the design of the coordination sites received little attention. The success was somewhat serendipitous and improved by keeping the metal content low. Understanding the importance of ensuring a well-defined bonding with the support enabled the preparation of the first industrially-amenable SACs, displaying high thermal stability and selective character in alkyne semi-hydrogenation^7^. The crystalline nature of the polymeric graphitic carbon nitride carrier, containing six-fold nitrogen-coordinating cavities intrinsic to the lattice, offers abundant anchoring points for metals and facilitates structural analysis by reducing the diversity of coordination structures.

In the last years, interest in tailoring the local environment of single atoms has grown intending to optimize the geometric and electronic properties as well as the associated reactivity of the resulting materials. Variation of the speciation of metal atoms on carbon supports enabled the development of the first stable heterogeneous catalyst for the sustainable production of vinyl chloride via acetylene hydrochlorination (Fig. 1, L15)^8^. Whereas initial attempts focused on gold single atoms supported on nitrogen-doped carbons due to their superior activity, the presence of the heteroatom in the support led to rapid deactivation due to the deposition of carbonaceous deposits. In contrast, platinum atoms are stable on activated carbons without the need of introducing nitrogen functionalities as binding sites, endowing them with unparalleled durability. Besides the chemical identity and arrangement of nearest-neighbor atoms, researchers are increasingly exploring the effects of tailoring the second coordination sphere and beyond (Fig. 1, L11)^9^.

The specific host and coordination environment determine the electronic structure of SACs. Since strong binding through ionic or covalent interactions is typically essential to firmly anchor single atoms, this can induce significant charge transfer. The cationic or anionic nature of the minority element can strongly influence its adsorption characteristics, having beneficial or detrimental effects in catalysis. To access unprecedented reactivity, the properties of SACs should not just be simple interpolations of those of the constituents. In single-atom alloys, the electronic structure typically exhibits a mean-field behavior. Recently emergent characteristics were demonstrated in AgCu alloys by weak wavefunction mixing between minority and majority elements^10^. The resulting narrowing of the *d*-band originated a free-atom like electronic structure for copper single atoms, permitting both ionic and covalent contributions to adsorbate bonding (Fig. 1, L12). Distinct catalytic behavior can also result from electronic dynamics. The coexistence of dynamically interconnected charge states between platinum atoms and ceria enabled a low-temperature reaction path for CO oxidation (Fig. 1, L13)^11^.

## Breakthroughs for sustainable chemistry

The potential of single-atom catalysts has been explored in diverse thermo-, electro-, and photochemical applications ranging from small-molecule activation to the construction of fine chemicals. Within this broad context, two areas promise revolutionary breakthroughs; the replacement of precious-metal-based catalysts in energy-related transformations and of molecular catalysts in organic synthesis. In the first category, the large-scale application of electrochemical routes for the production of fuels is contingent on the development of inexpensive and efficient catalytic materials (Fig. 1, L2). Exceptional performance has been demonstrated for iron, cobalt, and nickel atoms isolated as single atoms on nitrogen-doped carbons for the oxygen reduction^2^, hydrogen evolution^12^, and carbon dioxide reduction^13^ reactions. Research on single-atom photocatalysts is at an earlier stage than electrocatalysts, but also shows promising potential for contributing to solve the energy crisis (Fig. 1, L9)^14^.

The well-defined geometric and electronic properties created by the specific interaction of isolated atoms with host materials resemble those defined by ligands in molecular catalysts. The structural parallels present new opportunities to develop heterogeneous catalysts displaying competitive performance to state-of-the-art homogeneous analogs (Fig. 1, L8)^15,16^. The use of hosts providing a flexible coordination environment proved to be a critical factor in ensuring the catalyst stability, adapting to the specific requirements of the catalytic cycle^16^. While SACs are unlikely to provide a drop-in solution, these studies call for dedicated efforts to design low-nuclearity heterogeneous catalysts for the synthesis of complex organic molecules.

## From single atoms to low-nuclearity clusters

Single atoms are not the only entity of minority elements with the potential to attain 100% dispersion. Understanding the effects of nuclearity is crucial as adding or removing an atom can influence the properties in a non-scalable way as a result of both the quantum confinement of electrons in metals and distinctions in the interaction with the host material. However, both the stabilization of species of precise nuclearity and distinguishing between them poses an enormous challenge. The critical effects of adding or removing a single atom were evident for palladium catalysts synthesized by depositing precursors of the desired size onto a carbon nitride host containing cavities that can accommodate metal species of distinct size^17^. In the selective hydrogenation of alkynes, trimers displayed superior activity to dimers or isolated centers, whereas single atoms exhibited unmatched selectivity and stability in Suzuki coupling. Nuclearity effects were also pivotal in the promotion of an indium oxide catalyst with a palladium promoter for more efficient carbon dioxide hydrogenation^18^. Ensuring a surface palladium ensemble of fewer than three atoms was crucial to curtailing the competitive reverse water–gas shift reaction. Recently, the area of dual-atom catalysis, both homo- and heteronuclear, has attracted growing interest (Fig. 1, L10)^19^. The use of atomically dispersed clusters permits increased metal loadings and can increase the versatility by providing neighboring atoms of defined chemical identity.

## Frontiers in the design

Catalysts based on single atoms have long existed in different forms. However, the ability to visualize their presence in previously unknown systems has sparked enormous renewed interest in the past decade. The creativity of researchers, for example, towards the atomic design of host materials and the exploration of nuclearity trends, shows no boundaries. Nonetheless, it has surpassed the limits that can be resolved practically by state-of-the-art techniques such as aberration-corrected transmission electron microscopy imaging and X-ray absorption fine structure analysis (visualizing the environment is generally not feasible). The difficulty in experimentally verifying the structures has generated a growing reliance on computational methods mainly based on density functional theory to support hypotheses for the observed reactivity. Discoveries of breakthroughs in the catalytic performance of SACs compared to traditional heterogeneous catalysts convincingly demonstrate the strong potential of these materials in various applications, but further advances in both the controlled synthesis and characterization are required to move forward in the design. Concerning the synthesis of SACs, areas for improvement include the development of strategies to achieve ultra-high loadings and to precisely control the speciation and nuclearity of atomically-dispersed species, as well as a greater focus on scalable routes to accelerate commercialization (Fig. 1, L14)^20^. Tool development needs to focus on resolving the local environment with increasing precision, quantifying the real dispersion of SACs - as minority element centers may not be located on the external surface of host materials - and the separation between neighboring sites. Besides, an improved understanding and description of dynamic behaviors, especially under operating conditions, will be invaluable.
